# Effect of Puerperal Infections on Early Neonatal Mortality: A Secondary Analysis of Six Demographic and Health Surveys

**DOI:** 10.1371/journal.pone.0170856

**Published:** 2017-01-25

**Authors:** Saverio Bellizzi, Quique Bassat, Mohamed M. Ali, Howard L. Sobel, Marleen Temmerman

**Affiliations:** 1 World Health Organization, Department of Reproductive Health and Research, Geneva, Switzerland; 2 ISGlobal, Barcelona Ctr. Int. Health Res. (CRESIB), Hospital Clínic—Universitat de Barcelona, Barcelona, Spain; 3 Centro de Investigação em Saúde de Manhiça (CISM), Maputo, Mozambique; 4 ICREA, Barcelona, Spain; 5 World Health Organization, Western Pacific Regional Office, Manila, Philippines; 6 Department of Obstetrics and Gynaecology, Ghent University, Ghent, Belgium; Centre Hospitalier Universitaire Vaudois, FRANCE

## Abstract

**Background:**

Around 1.5 million annual neonatal deaths occur in the first week of life, and infections represent one of the major causes in developing countries. Neonatal sepsis is often strictly connected to infection of the maternal genital tract during labour.

**Methods:**

The association between signs suggestive of puerperal infection and early neonatal mortality (<7 days of life) was performed using Demographic and Health Surveys (DHS) data of six countries, conducted between 2010 and 2013. The population attributable fraction (PAF) was generated using the estimates on early neonatal mortality of a 1990–2013 systematic analysis for the Global Burden of Disease Study.

**Results:**

Signs of puerperal infection ranged from 0.7% in the Philippines to 16.4% in Honduras. Infection was associated with a 2.1 adjusted Risk Ratio (95% CI: 1.4–3.2) of early neonatal mortality. Around five percent of all deaths in the first week of life were attributable to signs suggestive of puerperal infections and varied from 13.9% (95% CI: 1.0–26.6) in Honduras to 3.6% (95% CI: 1.0–8.5) in Indonesia.

**Conclusions:**

Targeted interventions should be addressed to contain the burden of puerperal infections on early neonatal mortality. Consideration of the PAF will help in the discussion of the benefits of antenatal and perinatal measures.

## Introduction

Surviving the first days of life is a major challenge for neonates in low and middle-income countries (LMICs). Of the estimated 2.1 million annual neonatal deaths, three quarters occur in the first week of life [1]. Despite the rapid global reductions in infant child mortality in recent years, neonatal mortality has decreased at a slower pace. Consequently, neonatal mortality accounts for 44% of all child mortality rate in 2013 compared to 37.4% in 1990 [2].

Responsible for 22.7% of the total neonatal deaths [3], neonatal infections cause around 626,000 deaths annually [2], 99% of which occurring in LMICs [4].

Neonatal sepsis (with or without accompanying meningitis) is the most common infectious complication in this period, and may account for 34% of neonatal deaths in countries with neonatal mortality exceeding 44 per 1000 live births [5].

Up to 40% of infections leading to neonatal sepsis are transmitted at the time of birth, and are classified in early-onset (EOS) sepsis and late onset sepsis (LOS) [6].

EOS are typically due to the vertical transmission of Group B streptococci (GBS) [6] during the intrapartum period [7], and can manifest within the first 72 hours of life [8].

Puerperal infection is an infection of the genital tract occurring at labour or the postpartum period. It commonly presents with fever, pelvic pain and foul-smelling vaginal discharge [9], and has been associated with neonatal sepsis [10,11].

While puerperal infection accounts for 15% of all maternal deaths [12], no studies to our knowledge attempted to quantify its effect on neonatal survival. If significant, further attempts to accelerate reduction of neonatal mortality will need to focus on identifying and treating women with puerperal infections.

Using six Demographic and Health Surveys (DHS) with data on complications during delivery, we aimed to determine the association between puerperal infection and the risk of early neonatal mortality.

## Methods

### Study design

We searched the DHS publicly available surveys since 2010.We included the last live births of any mother in DHS with available information on mortality, obstetric complications during delivery and place of delivery.

Only six surveys met the search criteria amongst DHS conducted since 2010: Colombia 2010, Bangladesh 2011, Honduras 2011, Peru 2011, Indonesia 2012 and Philippines 2013. The DHS are comparable nationally representative surveys conducted in more than 80 countries worldwide since 1984 and collecting a wide range of population health data with strong focus on maternal and child health [13].

### Outcome, exposures and covariates

The outcome variable of this analysis was the occurrence of death in the first week of life (days 0–6, “early neonatal mortality”).

The DHS collected information on four obstetrical complications: prolonged labour, excessive bleeding, convulsions and high fever with foul smelling discharge.

The binary exposure variable of interest in our analysis was “high fever with foul smelling discharge” during delivery, suggestive of puerperal infection at the time of birth [14–16]. Our exposure variable definition did not include postpartum infection. We excluded cases with other obstetrical complications (i.e. prolonged labour, excessive bleeding and/or convulsions).

Covariates explored included maternal age (categorized in the three groups “15–24”, “25–34”, and “35–49”) and education (subdivided in “no education”, “primary”, “secondary”, and “higher”), wealth quintiles (“poor”, “middle”, and “rich”), parity (categorized in the five groups “0”, “1”, “2–3”, “4–5” and “>5”), and the binary place of residence split by “urban” and “rural” setting; we also derived the variable “place at delivery” based on the reported place and assistance at delivery: facility delivery was defined as any place other than delivering at home, at someone else’s home, or *en route* to a facility; deliveries at home with a skilled birth attendant (SBA) included respondents who answered that they delivered at home with a doctor, nurse, nurse midwife, or auxiliary nurse midwife.

### Study population

We pooled the six country datasets into one database containing 310,398 live births. However, DHS reported data on complications during delivery only for the last live births (n = 56,354) but not previous live births. As the objective of our analysis was to assess the association of puerperal infection with early neonatal deaths, we excluded all pregnancies complicated by convulsions, excessive bleeding and prolonged labour to eliminate their association with neonatal mortality. We also eliminated neonatal deaths occurring after the first week of life. Finally, we excluded multiple births as this is known to be associated with neonatal mortality. Our final study population resulted in 39,654 livebirths.

### Statistical analysis

The analysis was performed on the pooled dataset from 6 surveys using STATA 13.1 SE (StataCorp LP, USA) [17]. The pooled analysis took into account the survey design (weight, stratification and clustering) [18].

The country-specific prevalence of puerperal infectious complications without other obstetrical complications was calculated.

Considering the short and well defined risk period for the under-study outcome [19], the risk ratio (RR) and adjusted RR between early neonatal mortality and the “high fever with foul smelling discharge” complication with no other associated complications was estimated using generalized linear models. In the pooled analysis, the between-country heterogeneity was taken into account using random effects modelling and controlling for potential confounding effect using the selected covariates. P-values of <0.05 were considered significant.

Interaction between the exposure variable puerperal sepsis and the covariates maternal age, education, wealth, parity and place of delivery were tested.

Considering the wide difference in the prevalence of the exposure variable between five country-surveys and the Honduras survey, we performed a sensitivity analysis excluding the latter one.

Total annual early neonatal deaths by country were obtained from published estimates [20]. Population attributable fraction (PAF) of early neonatal deaths attributable to puerperal infections were calculated for each country with available data (Bangladesh, Colombia, Honduras, Indonesia and Peru): P(E)(RR-1)/[1+P(E)(RR-1)], where P(E) was the proportion of early neonatal deaths and RR the risk ratio of early neonatal mortality and the mother’s report of “high fever with foul smelling discharge”.

This PAF theoretically provides the proportional reduction in early neonatal deaths if puerperal infections were prevented or successfully managed preserving mother and child’s lives.

### Ethical approval

The datasets used in this study were obtained from the DHS program thanks to the authorization received to download the dataset on the website (https://dhsprogram.com/data/available-datasets.cfm).

## Results

Of the total 39,654 deliveries with singleton births in the six analyzed Demographic and Health Surveys (Bangladesh 2007, Colombia 2010, Honduras 2011, Indonesia 2012, Peru 2011 and Philippines 2013), 1,608 (4.0%) were complicated with signs suggestive of puerperal infection but without other obstetric complications. The percentage of women who reported signs of infection at delivery ranged from 0.7% in the Philippines to 16.4% in Honduras (Table 1).

**Table 1:** Number of live births, early neonatal deaths and puerperal infections in six low-and middle-income countries at different time points between 2010 and 2013.

*Country*, *survey years*	*N livebirths*	*Early neonatal deathsn (%)*	*Puerperal infection with no other obstetrical complications associated*, *n (%)*
Bangladesh 2011	3,418	72 (2.1)	38 (1.1)
Colombia 2010	10,051	104 (1.0)	123 (1.2)
Honduras 2011	6,682	86 (1.3)	1,095 (16.4)
Indonesia 2012	10,204	156 (1.5)	233 (2.3)
Peru 2011	5,596	60 (1.1)	92 (1.6)
Philippines 2013	3,703	47 (1.3)	27 (0.7)
Total	39,654	525 (1.3)	1,608 (4.0)

Age of mother, educational level, wealth quintiles, parity and place of delivery and place of residence were associated with puerperal infection (Table 2): youngest, poorest and mothers living in rural areas as well as *primigravidae* mothers were more at risk of developing signs of infection; on the other hand, higher education attainment was associated with low prevalence of signs of puerperal infection. As far as place and assistance at delivery is concerned, self-reported signs of complications decreased from health facility (4.8%) to home with SBA (4.5%) up to as low as 1.5% at home without SBA (Table 2).

**Table 2:** Maternal characteristics in deliveries with no complications and in deliveries with infectious complications among 39 654 livebirths occurring in nine low and middle-income countries at different time points between 2010 and 2013.

		*All deliveries*	*Deliveries with no complications*		*Deliveries with infection with no other complications*		
		N	n	(%)	n	(%)	Χ^2^
Maternal Age (years)	15–24	12,773	12,113	(94.8)	660	(5.2)	<0.001
	25–34	18,185	17,492	(96.2)	693	(3.8)	
	35–49	8,696	8,441	(97.1)	255	(2.9)	
Parity	0	12,510	11,870	(94.9)	640	(5.1)	<0.001
	1	9,121	8,838	(96.9)	283	(3.1)	
	2–3	9,116	8,852	(97.1)	264	(2.9)	
	4–5	5,838	5,633	(96.5)	205	(3.5)	
	>5	3,069	2,945	(96.0)	124	(4.0)	
Education	No education	2,278	2,205	(96.8)	73	(3.2)	<0.001
	Primary	13,745	12,905	(93.9)	840	(6.1)	
	Secondary	17,582	16,995	(96.7)	587	(3.3)	
	Higher	6,046	5,938	(98.2)	108	(1.8)	
Wealth Index	poor	14,546	13,979	(96.1)	567	(3.9)	<0.001
	middle	13,221	12,851	(97.2)	370	(2.8)	
	rich	11,887	11,483	(96.6)	404	(3.4)	
Place of residence	Rural	20,376	19,403	(95.2)	973	(4.8)	<0.001
	urban	19,278	18,643	(96.7)	635	(3.3)	
Place of delivery	home	8,586	8,461	(98.5)	125	(1.5)	<0.001
	home with SBA	4,470	4,270	(95.5)	200	(4.5)	
	health facility	26,420	25,145	(95.2)	1,275	(4.8)	

Both unadjusted (RR 1.6; 95% CI: 1.1–2.3) and adjusted logistic regression (RR 2.1; 95% CI: 1.4–3.2) showed significant association between the risk of early neonatal death and puerperal infection in the pooled analysis (Table 3).

**Table 3:** Pooled and country RR of early neonatal death in relation to the effect of puerperal infections.

*Country*, *survey years*	*RR (95%CI) unadjusted*	*RR (95%CI) adjusted*^*a*^	
Pooled	1.6 (1.1–2.3)	2.1	(1.4–3.2)
Bangladesh 2011	7.1 (2.3–21.8)	7.8	(2.3–26.8)
Colombia 2010	4.5 (1.4–15.4)	5.4	(1.6–17.9)
Honduras 2011	1.8 (1.1–3.4)	2.0	(1.1–3.5)
Indonesia 2012	2.1 (1.0–5.2)	2.6	(1.1–6.6)
Peru 2011	4.4 (1.4–14.0)	5.2	(1.6–17.2)
Philippines 2013	\ \	\	\

^a^ Adjusted for place of delivery, sex of the child, wealth, age, parity, education, and country as cluster random effect in the pooled analysis.All country-specific analyses had significant associations; for Philippines DHS, no deaths in complicated pregnancies were present. A sensitivity analysis removing Honduras on account of its much higher prevalence of infection signs at delivery from the logistic regression increased the risk of early neonatal death to 3.1 (95% CI: 1.5–4.5).

We did not find significant interactions between puerperal sepsis and the covariates maternal age, education, wealth, parity and place of delivery for risk of early neonatal mortality.

In the five countries with available data, 5,756 out of 124,524 neonatal deaths in the first week of life were attributable to puerperal infections corresponding to 4.6 percent of the total early neonatal deaths. These ranged from 13.9% (95% CI: 1.0–26.6) in Honduras to 3.6% (95% CI: 1.0–8.5) in Indonesia (Table 4).

**Table 4:** Total early neonatal deaths (N)^a^, Population Attributable Fraction with 95% Confidence Interval, and estimated number of early neonatal deaths due to puerperal infections per country.

*Country*, *survey years*	*Total early neonatal deaths*^*a*^	*Population attributable fraction (95% CI)*^*b*^	*N early neonatal deaths due to puerperal infection*
Bangladesh 2011	60,643	5.1 (1.0–11.3)	3,092 (606–6,853)
Colombia 2010	4,735	5.4 (1.0–12.4)	256 (47–587)
Honduras 2011	1,912	13.9 (1.0–26.6)	266 (19–508)
Indonesia 2012	52,434	3.6 (1.0–8.5)	1,888 (524–4,457)
Peru 2011	4,800	5.3 (1.0–12.3)	254 (48–590)

^a^ calculation based on rates published in the publication ***“Wang H*. *et al*, *Global*, *regional*, *and national levels of neonatal*, *infant*, *and under-5 mortality during 1990–2013*: *a systematic analysis for the Global Burden of Disease Study 2013*,** Lancet. 2014 Sep 13;384(9947):957–79.***”***

^b^ adjusted for place of delivery, sex of the child, wealth, age, education, parity, wanted pregnancy, rural\urban residence.

## Discussion

This secondary analysis of data collected in six very distinct countries through DHS found an overall high prevalence (up to 4.0%) of obstetrical infectious symptoms among pregnant women. Importantly, presenting such symptoms without other associated obstetric complications was significantly associated with the occurrence of early neonatal mortality (RR 2.1; 95% CI: 1.4–3.2). This suggests to around one in every twenty early neonatal deaths could be avoided if puerperal infections were appropriately managed. Applying this calculated attributable fraction to our current estimates of neonatal mortality in the 6 countries surveyed, suggests up to 5,756 out of 124,524 early deaths could be prevented.

Estimating the real prevalence of puerperal infection in developing countries presents many challenges, including the lack of a well-accepted case-definitions [21], lack of access to health facilities [21] as well as the frequent onset of symptoms after discharge with lack of postnatal care with inadequate diagnosis and underreporting [22].

Individual studies from developing countries have reported puerperal infection incidence estimates ranging from 0.1% to 10% of deliveries [23,24], and this complication appears to be up to 10 times more common after caesarean sections [25].

Epidemiological estimations vary widely due to the coexistence of other infections, such as HIV, or clinical, cultural, religious and hygienic practices during delivery [22].

Demographic and Health Surveys are often the only source of maternal and child health information available for many developing countries and are commonly considered high-quality surveys [26] that utilize standardized methodologies, thus reducing the risk of inter-country variation. [27] However, one must take into consideration that DHS were compared across 6 countries at various times after 2010. The findings may vary in countries with large differences from those countries represented here.

Self-reported maternal complications in the different countries appear low, ranging from 2% to 5%, for all countries with the exception of Honduras, which reported maternal complications in over a fifth of all mothers (21.6%). These data need to be interpreted with caution and this was the rationale for conducting a sensitivity analysis without including the data from Honduras, which significantly increased the strength of the association hypothesised.

Another limitation relates to the problems in accuracy associated with maternal recall of complications like infection [28,29]. The reliability of self-reported maternal morbidity may vary according to a variety of aspects, including cultural and religious backgrounds, socioeconomic status, or how the mother interprets and reports signs, symptoms and their severity as well as health workers’ skills to recognize and communicate to the mother. Indeed, mother’s perceptions may be ranked very differently from those of medical professionals [30].

We adjusted our analysis for socio-economic variables and place at delivery, which are well documented to be linked with both early neonatal mortality and puerperal infections; however, we were not able to consider further potential confounding factors such as the anemia and HIV status in pregnant women [31–33].

An additional limitation is that maternal complications were reported after the neonatal death had occurred (or not occurred), which could have led to information bias. This may lead to an overestimation of any associations between puerperal infection and early neonatal death.

Additionally, another limitation relates to the lack of information on women who died during delivery, which is both likely associated with an increased risk of maternal morbidity and complications but also with an increased risk of early neonatal deaths. However, the proportion that died, having signs of puerperal infection and an affected newborn may be very small.

A further limitation may stem from the fact that women are less prone to report information on non-surviving children, leading to problems on the estimation of the actual incidence of the early neonatal mortality [34,35].

Furthermore, the population attributable fraction was calculated assuming that all neonatal deaths associated with maternal high fever with foul smelling discharge in absence of other maternal complications were cases of neonatal sepsis. This is likely to be an important underestimation, as despite several studies having identified maternal pyrexia as a clear risk factor for neonatal sepsis [11,36,37], not all early neonatal confirmed infections have this maternal antecedent.

To our knowledge, this is the first large study attempting to determining the association of puerperal infections with early neonatal mortality. Our analysis revealed maternal high fever with foul smelling discharge presumed to be puerperal infection was strongly associated with neonatal mortality, presumed to be neonatal sepsis. However, our findings may underestimate the association since we did not consider all early deaths where signs of infection at delivery where associated with other obstetrical complications.

Moreover, the impact could be even worse in settings with higher neonatal mortality where the incidence of infections is more prominent.

Several risk factors have been clearly studied and associated with the occurrence of maternal infection and include caesarean section [38], episiotomy [39], increased number of vaginal examinations [39], as well as hygiene and socio-economic conditions [40].

To contrast the burden of puerperal infections on neonatal mortality, a series of interventions should be enhanced aiming to minimize infection in the peri-partum period using antisepsis measures and antibiotics prophylaxis on one side, and to reduce the risk of maternal infection through antenatal care and nutritional supplementation.

Without adequately tackling the burden of maternal infections during pregnancy, it will be challenging to improve the survival of neonates in settings where both diagnostic and therapeutic tools are scarce, and cannot guarantee and early diagnosis and treatment.
